# Uterine Fibromata

**Published:** 1893-01

**Authors:** J. M. Baldy

**Affiliations:** Professor of Gynecology in the Philadelphia Polyclinic, Surgeon to the Gynecean Hospital


### UTERINE FIBROMATA. REMOVAL OF TWENTY-SEVEN WITH TWO DEATHS.*

BY J. M. BALDY, M. D.,

Professor of Gynecology in the Philadelphia Polyclinic, Surgeon to the Gynecean Hospital.

It is not many years since it was almost universally taught that fibroid tumors of the uterus were harmless, and that their presence might with impunity be ignored. Either women or the character of these neoplasms have changed since those times, or there must have been a fearful negligence and indifference to the welfare of many of the sufferers. Possible it is that the profession, being only too fully awake to the danger of the only rational treatment, preferred rather to shirk than to face the issue. However this may be, the tremendous strides of abdominal surgery have put this disease, together with many others, within the number of affections which can be cured with comparative ease.

The symptoms to which the affection gives rise are variable and are dependent to a great extent, with possibly a single exception, upon the size and shape which the growth may assume. The fibroid tumor in itself, as long as it remains a true fibroma, will occasion little trouble other than the induction of uterine

*Read before the Philadelphia College of Physicians, Nov. 2, 1892.

hemorrhage to a greater or less extent. As it grows its presence will sooner or later induce irritation of the peritoneal surfaces, thus giving rise to considerable pain and oftentimes distension. The irritation may even amount to an inflammation which is apt to be kept up or relighted frequently or not according to the amount of exposure to determining causes. Pressure within the pelvis especially gives rise to very distressing symptoms, even outside of the pain which it causes. The bladder is most frequently irritable and incapacitated for holding the urine for any great length of time. It has been my experience to find this organ many times in a condition of chronic inflammation, the urine being loaded with pus. The bowels are mostly constipated, due to a great extent to the mechanical obstruction caused by the pressure, and it is a matter of surprise to me at times when handling these tumors, how any fæcal matter whatever has been able to pass the pelvic brim. Patients carrying a tumor of this class are liable to repeated attacks of peritonitis, attacks which are not always sufficiently violent to endanger life materially, but which render the woman an invalid for weeks and often months. It would seem at first blush that the tumor of a woman who had suffered from one or more attacks of peritoneal inflammation must of necessity be adherent to surrounding parts. This is not the case, however, excepting where the inflammation is septic or specific. A septic or specific inflammation of this serous membrane, almost invariably leaves adhesions in its train; a simple traumatic peritonitis is apt to clear up without such serious consequences. Although fibroid tumors are apt oftentimes to be complicated by diseased uterine appendages and are thus open to the chances of an adhesive inflammation, yet it is a fact that the majority, even of the complicated ones, are accompanied by few if any adhesions, even after repeated inflammatory attacks.

The amount of suffering attendant upon these growths is as a rule in direct ratio to the complications and has little or nothing to do with the size, unless that has become enormous. The sufferings of a woman carrying such a growth as large as a full term gravid womb, is frequently not nearly so great as those of one with a tumor which barely rises out of the pelvis.

Much has been written concerning the organic changes in various organs caused by the presence of fibroid tumors. These changes are as often mythical as not. It has occurred to me to find cystitis and more frequently a thickened parietal peritoneum, but otherwise in the twenty-seven uterine fibroids which I have removed, the other organs of the patient have been healthy. In two or three of them I have found albumin in the urine, but it was evidently due to pressure, as it cleared up in all but one case almost at once after operation. In all of my patients, with this one exception, the kidneys have been healthy. The ureters have never been observed in an abnormal condition; the bowels have been normal and the other abdominal organs have remained unaffected. I find myself therefore unable to subscribe to the oft-repeated cry of the dangers of irreparable organic changes in the abdominal organs due to the presence of fibroid tumors of the womb. It has been my lot, however, to find frequent sarcomatous change taking place in the tumors themselves, tumors which had formerly been apparently perfectly healthy. Diseased uterine appendages accompanying the growth have been observed very frequently, in fact in the majority of those which have come under my notice; pus tubes, hydrosalpinx, old chronic salpingitis, with great hyperplastic enlargement of the ovaries, ovarian cysts, ovarian abscesses, have been the most frequent complications. One case of broad ligamentous tumor, or more correctly uterine tumor, which had grown into and between the folds of the broad ligament, was complicated by adhesions to several coils of intestines; these being released and the tumor left *in situ* on account of the risks of the operation, the symptoms all subsided, and there has never been a return of any of them, it being now more than a year and a half since the operation.

The old methods of treating this disease have not much to recommend them. As applied to many cases of uterine fibroma, they will probably answer as well as anything, because many cases require no treatment at all; in other words the mere presence of a fibroid tumor is no warrant for dosing the patient with purgatives, ammonia, ergot, hydrastis, electricity and similar remedies. It must not be expected that the tumor can be gotten rid of short of surgical methods, and these are to be reserved for special cases. When it comes to dealing with symptoms, however, these remedies all have their place and are capable of doing much good, provided they are used judiciously. Rest in bed, purgation, counter-irritation, hot douches, electricity, etc., will frequently relieve the pain which is most often due to peritoneal irritation and which should be treated on general principles. The curette, especially in small growths, ergot, hydrastis, atropia, electricity and similar remedies come into play for the hemorrhages and will temporarily control the bleeding. If one is able to accomplish this much, control the pain and hemorrhage, he has often accomplished all that the patient desires and it would be hard to persuade them into having an operation performed. If the inflammatory attacks are not recurring, and the hemorrhage is not excessive, provided the tumor is not so large as to cause trouble by its size, the patient may rest content with her present condition, especially if she be approaching the menopause. It by no means follows as has been so often taught, that most of these neoplasms cease to grow at this period of life. On several occasions has it been found necessary in my experience to remove the uterus, together with the growth in women forty-eight or fifty years of age. Yet, an occasional case occurs in which the change of life ends the period of suffering and the growth ceases to cause active symptoms. In view of this fact if the woman is near the time of her change, it may be worth her while to wait, provided that her symptoms are not too distressing. Each of these cases must be a law unto itself as far as the consideration of surgery is concerned, and yet fairly accurate lines can be laid down along which it will be safe to advise any given patient.

1. All rapidly growing fibroid tumors in young women (before thirty-five) should be removed; and many of the same kind as late as forty years of age.

2. All cases in women under forty, where there is such loss of blood as to enfeeble the general health, and which is not readily controlled by treatment.

3. All cases under forty in which there are frequent recurring attacks of peritonitis.

4. Cases which have gone several years past the menopause with excessive uncontrollable bleeding or recurrent attacks of peritonitis.

Such advice as this would a few years ago have seemed unwise and unsafe, but in veiw of our present successes in the surgery of such growths we are justified in adopting more radical measures than formerly. No one can doubt but that a woman is better and safer without a fibroid tumor than with one, and therefore the only questions worth considering are with how much safety can they be removed and is there any surer or safer method of removing them than by extirpation ?

Oöphorectomy has been proposed and practiced in times past for the purpose of accomplishing this object, but has proven itself both unsatisfactory and unreliable, besides being almost as dangerous as the removal of the tumor. The operation excepting for small pelvic tumors has passed out of use. The claims made for electricity, as far as the removal of fibroid tumors is concerned, have proven, as predicted, fallacious, and to-day none but the most enthusiastic electro-gynecologists are making any claims in that direction, and these claims are made invariably without proof. The removal of a healthy non-adherent fibroma of the womb should be and is attended with but a small percentage of danger. Of the twenty-seven tumors of this character which it has been my fortune to remove by the extra-peritoneal method but two (about 7 per cent.) have died. In neither of these cases could the fault be placed on the operation, but on the operator and the previous treatment. The first death was that of a colored woman with a tumor which weighed much over twenty pounds. During the operation a towel (which had not been prepared for the purpose) was placed in the abdominal cavity to hold the intestines in place, while the necessary steps for treating the pedicle were carried out. This towel infected the peritoneal cavity and the woman died five days later ofpurulent peritonitis. The second patient was a white woman whom I had seen three years before in good health, with a healthy movable tumor. At the time of the operation she was an emaciated, bedridden woman with a rapid, feeble pulse, a high temperature and deeply septic. The tumor was fixed by adhesions, and a sinus which discharged pus freely was found on the outside of the left labium major and pus was flowing from the vagina. The intestines and omentum proved to be adherent to the tumor, the tumor was adherent to the whole pelvis, both ovaries contained pus and the external sinus opened into the left ovary, and both ovaries and tubes were universally adherent. When the uterus, tumor, Fallopian tubes and ovaries were all freed, delivered and removed, there remained sinuses into the vagina and into the cellular tissue about the vaginal sheath. These terrible complications all followed a course of treatment by electro-puncture in the hands of an expert. That the patient died of purulent peritonitis was surely no discredit to hysterectomy. With these two exceptions all the tumors of this character which I have removed by the method which I almost invariably adopt have had as easy convalescence as a patient who has had an operation for a simple ovarian cyst would have had.

The method used has been that known as the extra-peritoneal treatment of the stump. In this operation, the tumor is turned out upon the abdomen and a wire clamp is placed about the base of the neoplasm in order that it may constrict it and control the bleeding. A transfixion pin is then placed above the wire to keep it from slipping up or the stump from being pulled down through the grasp of the wire. The stump is then fixed in the lower angle of the incision and the abdominal wound closed. Whenever there is an inclination for the stump to bleed, the wire is screwed tighter and any danger of hemorrhage is thus avoided. The advantages of this plan of treatment are first that the stump is under perfect control, and if it shows any signs of bleeding it can be checked instantly by screwing up the wire; secondly, if there be any septic matter in the cervical canal or about the stump it is outside of the abdominal cavity and may suppurate with safety; thirdly, all raw or denuded surfaces are outside of the peritoneal cavity and there is no chance for adhesions to form between them and the intestines.

The disadvantages claimed for this method of treating the stump are that the free action of the bladder is interrupted and that hernias are apt to follow at the point where the stump was originally fixed. The bladder certainly is irritable as long as the wire is kept on the stump, but this is corrected as soon as the stump has sloughed away or the wire is taken off. In but two cases have I seen hernia. In the one patient it appeared at the site of a drainage tube which had been used and was more than an inch above the pedicle and could consequently not be put down to anything but drainage. The second hernia appeared at the site of the old stump, The operation had been done in the case of a large sarcomatous uterus which was cut away and the stump treated outside the peritoneal cavity. As a matter of course there was not good union between the sarcomatous tissue of the stump and the surrounding tissues, and I was not at all surprised when the woman returned with a hernia. That the hernia did not appear in any of the other cases is pretty fair proof of the fallacy of the criticism. The dangers of septic peritonitis from a sloughing stump is a familiar battle-cry with those who are opposed to the method of treatment. Such a cry must be born of lack of experience; in no single case was there any such accident, although many of the stumps sloughed; in fact it is this danger of septic peritonitis that is avoided by the above method of treatment of the stump.

There is an occasional fibroid tumor of the uterus that cannot be brought out of the abdominal wound so as to place a wire about its base. This is caused by the fact that the growth has split open one or both broad ligaments and has developed between its folds. In these cases, it becomes necessary, if the tumor is to be removed, to tie off the broad ligaments and secure both the ovarian and uterine articles; in which case it is probably best to drop the stump back into the abdominal cavity or remove it entirely. However advantageous and easy this method may be in skilled hands, experience has taught me that as yet the extra-peritoneal method has proven the safest and surest and the time has not yet come for substituting for it other methods which have not been fully tried and matured. For the present at least, a beginner in hysterectomy should think of but one method of treating the stump (the extra-peritoneal) where that method is practicable, and as a matter of fact in the vast majority of cases it is practicable. It is both the easiest and safest method to begin with and if, after mature experience, one wishes to deviate and adopt a method which will give a less prolonged convalescence, he will find, if he has skilled fingers and a cool head, some of the intra-peritoneal methods now being rapidly developed both comparatively safe and sure, but much harder of performance and with many more elements of danger.

